# Magnetically Actuated Soft Microrobot with Environmental Adaptative Multimodal Locomotion Towards Targeted Delivery

**DOI:** 10.1002/advs.202406600

**Published:** 2024-09-24

**Authors:** Qingwei Li, Fuzhou Niu, Hao Yang, Dongqin Xu, Jun Dai, Jing Li, Chenshu Chen, Lining Sun, Li Zhang

**Affiliations:** ^1^ Robotics and Microsystems Center School of Mechanical and Electrical Engineering Soochow University Suzhou 215000 China; ^2^ School of Mechanical Engineering Suzhou University of Science and Technology Suzhou 215000 China; ^3^ Department of Orthopedic Surgery The Second Affiliated Hospital of Soochow University Suzhou Jiangsu 215004 China; ^4^ Department of Ultrasound The Second Affiliated Hospital of Soochow University Suzhou Jiangsu 215004 China; ^5^ Department of Mechanical and Automation Engineering The Chinese University of Hong Kong Hong Kong NT 999077 Hong Kong

**Keywords:** environmentally adaptive, magnetically actuated, multimodal locomotion, soft microrobot, targeted navigation

## Abstract

The development of environmentally adaptive solutions for magnetically actuated microrobots to enable targeted delivery in complex and confined fluid environments presents a significant challenge. Inspired by the natural locomotion of crucian carp, a barbell‐shaped soft microrobot (MBS^2^M) is proposed. A mechano‐electromagnetic hybrid actuation system is developed to generate oscillating magnetic fields to manipulate the microrobot. The MBS^2^M can seamlessly transition between three fundamental locomotion modes: fast navigation (FN), high‐precision navigation (HPN), and fixed‐point rotation (FPR). Moreover, the MBS^2^M can move in reverse without turning. The multimodal locomotion endows the MBS^2^M's adaptability in diverse environments. It can smoothly pass through confined channels, climb over obstacles, overcome gravity for vertical motion, track complex pathways, traverse viscous environments, overcome low fluid resistance, and navigate complex spaces mimicking in vivo environments. Additionally, the MBS^2^M is capable of drug loading and release in response to ultrasound excitation. In an ex vivo porcine liver vein, the microrobot demonstrated targeted navigation under ultrasound guidance, showcasing its potential for specialized in vivo tasks.

## Graphical abstract

A magnetic barbell‐shaped soft microrobot (MBS^2^M) is proposed inspired by the natural locomotion of crucian carp with the advantages of environmental adaptation, flexible movement, precise positioning, and multifunctionality. The proposed microrobot provides a “all‐in‐one” solution for sophisticated tasks in complex environments, which has excellent application potential and is expected to play an important role in biomedical applications such as drug delivery, detection, and diagnosis in vivo.

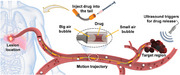

## 1 Introduction

Minimally invasive targeted therapy using magnetically actuated microscale robots (MAMR) in vivo has gained increasing recognition.^[^ ^1^, ^2^, ^3^, ^4^, ^5^ ^]^ Extensive research has focused on optimizing the design, fabrication, functionalization, actuation, control strategy, and application scenarios of MAMR for transforming them from laboratory studies to clinical trials. However, precisely controlling MAMR to reach target sites and perform therapeutic tasks in complex or confined fluid environments such as blood vessels remains a significant challenge in current clinical applications.^[^ ^6^, ^7^, ^8^, ^9^, ^10^, ^11^, ^12^, ^13^ ^]^ This complex and systematic challenge necessitates specific MAMR design and corresponding magnetic actuation source.^[^ ^14^, ^15^, ^16^, ^17^, ^18^, ^19^, ^20^, ^21^ ^]^ In order to achieve minimally invasive targeted in vivo therapy, MAMR must possess not only excellent motility, but also the ability to adapt different environments. It is necessary to enable efficient and accurate navigation, as well as reasonable adaptability to restricted spaces.^[^ ^1^, ^10^, ^22^, ^23^, ^24^, ^25^, ^26^ ^]^ Furthermore, the functional structure of MAMR to confer both loading and releasing capacity should be elaborately designed.^[^ ^9^, ^27^, ^28^, ^29^, ^30^, ^31^, ^32^ ^]^ Magnetic actuation sources considering suitable actuation strategy for MAMR also need to be developed. Moreover, the MAMR's motion control, navigation strategies, and detailed manipulation to accomplish specific tasks require further exploration.

MAMRs can be classified as either rigid or flexible on the basis of their ability to deform plastically. Rigid MAMR, which predominate over their flexible counterparts, typically consist of magnetic particles^[^ ^10^, ^14^, ^15^, ^18^, ^33^ ^]^ (mainly NdFeB) or rigid scaffolds made of paramagnetic materials such as Ni,^[^ ^34^ ^]^ Fe_3_O_4_, and Fe_2_O_3_.^[^ ^20^ ^]^ Rigid magnetically actuated microrobots demonstrate facile controllability and precise locomotive capabilities. However, they suffer from poor biocompatibility^[^ ^33^, ^34^, ^35^ ^]^ and adapt poorly to changing environments, making them potentially hazardous when handling tasks in complex or confined natural channels in vivo and prone to damage when interacting with soft tissues. To address these limitations, soft materials such as hydrogels;^[^ ^36^, ^37^, ^38^ ^]^ polymers;^[^ ^39^, ^40^, ^41^, ^42^, ^43^ ^]^ polydimethylsiloxane (PDMS);^[^ ^44^, ^45^ ^]^ GelMA;^[^ ^46^ ^]^ Ecoflex;^[^ ^47^, ^48^ ^]^ elastomers;^[^ ^49^ ^]^ ferrofluids;^[^ ^22^ ^]^ piezoelectric compounds;^[^ ^39^ ^]^ slime;^[^ ^50^ ^]^ and materials derived from natural objects, such as Magnetospirillum magneticum,^[^ ^51^ ^]^ bacteria,^[^ ^52^ ^]^ neutrophils,^[^ ^53^ ^]^ diatoms,^[^ ^9^ ^]^ sperm cells,^[^ ^54^ ^]^ proteins,^[^ ^55^ ^]^ and flagellated cells^[^ ^56^ ^]^ have been employed to construct flexible MAMR capable of more versatile motion and deformation abilities. These materials also offer superior biocompatibility, making them ideal for in vivo applications.

In recent years, bio‐inspired structures, materials, behaviors, and interactions have provided new design paradigms, actuation mechanisms, and system architectures for flexible MAMR (f‐MAMR). Numerous bio‐inspired f‐MAMR studies have incorporated rigid magnetic components within their actuation modules or internal structures with variable stiffness,^[^ ^57^ ^]^ enabling adaptation to surroundings and safe interaction with organisms.^[^ ^8^, ^9^, ^10^ ^]^ In general, these rigid magnetic components are embedded magnetic particles to guide the deformation of f‐MAMR.^[^ ^57^, ^58^, ^59^, ^60^, ^61^, ^62^, ^63^, ^64^, ^65^ ^]^ These works not only inherit advantages of f‐MAMR but also possess distinct motion characteristics and diverse motion patterns. Although extant bio‐inspired f‐MAMR possess unique characteristics and advantages, the motion efficiency^[^ ^58^, ^60^ ^]^ and accuracy^[^ ^57^, ^59^ ^]^ of microrobots require improvement, and their adaptability to the environment remains limited. Some microrobots rely on external environmental constraints^[^ ^61^ ^]^ and friction^[^ ^49^, ^62^, ^63^, ^64^ ^]^ for mobility or cannot traverse within narrow spaces.^[^ ^65^ ^]^ Moreover, integrating multiple beneficial functions into a systematic platform remains difficult.

**Figure 1:**
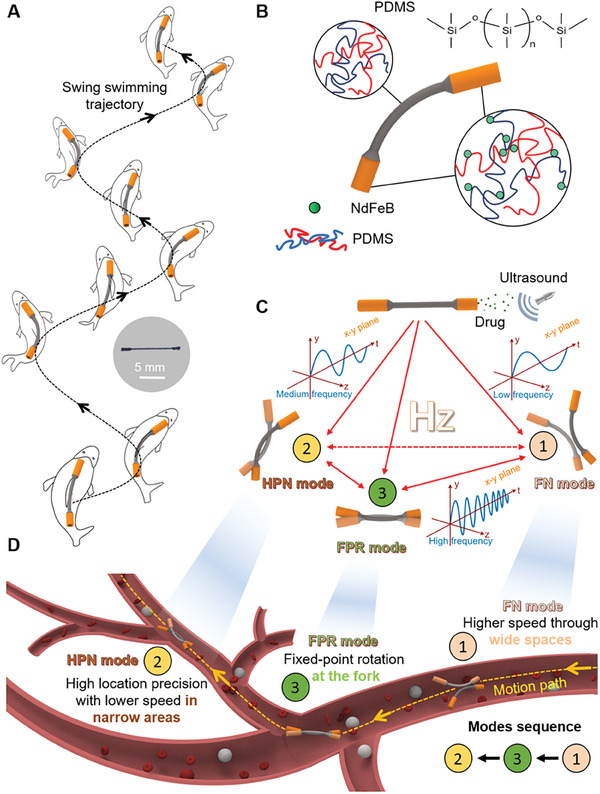
Schematic of the magnetic barbell‐shaped soft microrobot (MBS^2^M). A) Bioinspired design based on the locomotion of crucian carp. The illustration depicts a representative image of the fabricated MBS^2^M. B) Components comprising each part of the MBS^2^M. C) Interconversion between different locomotion modalities under multimode control strategies. D) Multimodal control guidance diagram for targeted drug delivery in a blood vessel.

In nature, crucian carp can quickly shrink its body into a C‐shape to achieve swift propulsion and smooth steering and modulate swinging frequency to adjust speed. Here, we propose a magnetic barbell‐shaped soft microrobot (MBS^2^M) to simulate the behavior of crucian carp (**Figure** **1**A) with the advantages of flexible movement, precise positioning, multifunctionality, and environmental adaptation. This microrobot is composed of a magnetic head, a magnetic tail, and a flexible body, resembling a barbell (Figure 1B). The underlying material of the MBS^2^M is PDMS, which endows the microrobot with high flexibility and deformability. The magnetic particles (NdFeB) are embedded within the tail and head propel and can exert the MBS^2^M's deformation by external magnetic field. Furthermore, we devised a mechano‐electromagnetic hybrid actuation (MEHA) system integrating an electric rotating platform and electromagnetic coil enabling programmatic generation of an oscillating magnetic field to propel the microrobot. Based on the MEHA system, the MBS^2^M can seamlessly transition between three fundamental locomotion modes: fast navigation (FN) mode, high‐precision navigation (HPN) mode, and fixed‐point rotation (FPR) mode by tuning the oscillation frequency of the actuation magnetic field. Each mode has unique locomotion performance (Figure 1C). In FN mode, the low driving magnetic field frequency and large oscillation amplitude enable the MBS^2^M rapidly advanced, which can effectively augment its targeted mobility and maneuverability. Moreover, the microrobot demonstrated the ability to make sharp turns in confined environments. In HPN mode, stable movement, and high positioning accuracy are conducive to directional drug delivery. Additionally, forward and backward movement without turning were demonstrated in the HPN mode, making it ideal for navigating narrow channels that can return to the foregone right position after a wrong‐way movement. In FPR mode, the high driving magnetic field frequency yielded small amplitude oscillations, enabling rotational manipulation in a fixed position that well suited for microrobot orientation adjustments. These locomotion modalities impart MBS^2^M's versatile environmental adaptability and movement capabilities. The MBS^2^M experimentally demonstrated versatility in traversing narrow channels, viscous media, surmounting obstacles, overcoming its inherent gravity, overcome the resistance of low viscosity and low flow rate fluids, and navigating complex spaces analogous to natural cavities. These physiological cavities (such as blood vessels, brain ventricles, etc.) typically exhibit branches of varying sizes and complex internal microenvironments. As shown in Figure 1D, we simulated targeted drug delivery through a blood vessel using composite control across multiple modes. To further validate the feasibility of this targeted delivery strategy, we conducted real‐time targeted navigation of the MBS^2^M in the ex vivo porcine hepatic portal vein under ultrasound imaging guidance. We envision that the capacity of the MBS^2^M to conduct multimodal locomotion in intricate, confined spaces could enable in vivo targeted delivery with substantial future biomedical applications.

## 2 Results

### 2.1 Fabrication and Characterization

**Figure 2:**
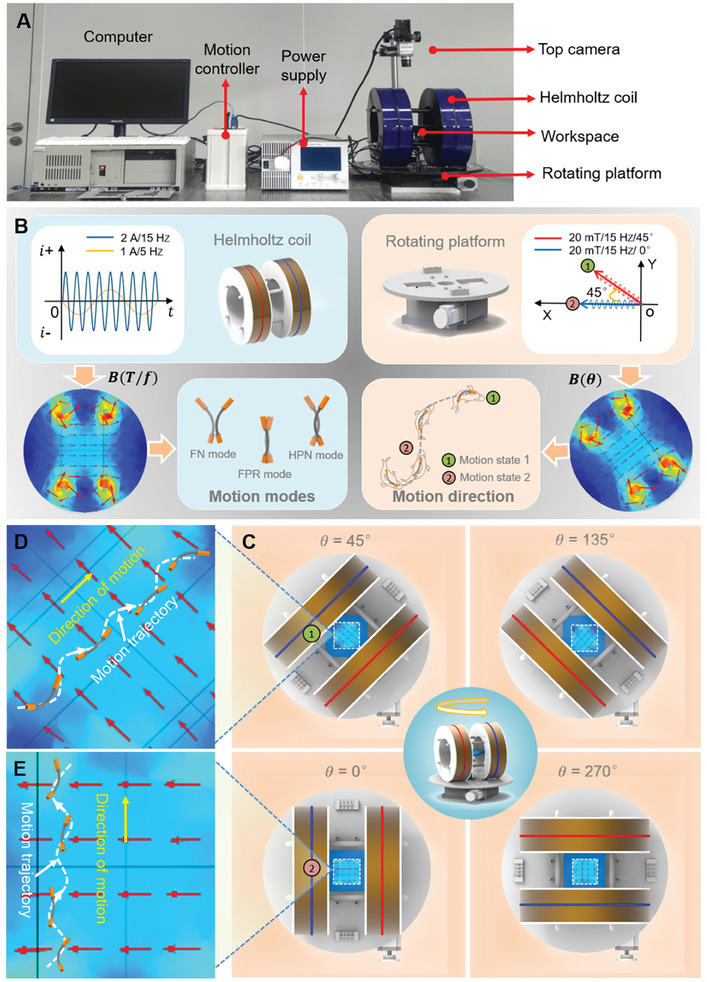
The MEHA system for actuating the MBS^2^M. A) Overall actuation system setup. B) Schematic of the MEHA system control MBS^2^M motion. C) Schematic of the adjustment of the magnetic field direction using an electric rotating platform. D,E) Modulating the orientation of the magnetic field influences the movement direction of the MBS^2^M. The direction of the magnetic field is denoted by a red arrow.

We developed a mechano‐electromagnetic hybrid actuation (MEHA) system to propel the MBS^2^M (**Figure** **2**A). The system comprises an actuation module, a computer, a programmable current source (APS‐1102A, Gwinstek, China), a motion controller, a CCD camera (XCD‐SX90, SONY, Japan). The actuation module is the core equipment of the MEHA system, composed of Helmholtz coils, an electric rotating platform (Y200RA400, Beijing Jiangyun Photoelectric Equipment Co., LTD, China). Helmholtz coils generate a magnetic field to control the motion modes of the MBS^2^M, while the electric rotating platform controls the direction of the generated magnetic field, thereby controlling the motion direction of the MBS^2^M. The MEHA system controls the MBS^2^M as shown in Figure 2B.

When a sinusoidal alternating current is applied to the Helmholtz coil, by adjusting the magnitude and frequency of the current, a driving magnetic field with an intensity of 0–100 mT and a frequency of 1–200 Hz can be generated within the square working space (100 × 100 mm). This driving magnetic field is essentially an oscillating magnetic field, as shown in Figure S1A (Supporting Information). The generated magnetic field is uniform within the workspace at any given moment, whose frequency mirrors that of the excitation current alterations and the magnetic flux density scales proportionally to the current's magnitude. Dynamic simulation of the magnetic field's spatiotemporal evolution is shown in Figure S1B and Movie S1 (Supporting Information). Additionally, the combination of the Helmholtz coil mounted on the electric rotating platform can generate an oscillating magnetic field along any direction in the plane (Figure 2C and Movie S1, Supporting Information), thereby controlling the direction of the MBS^2^M's movement (Figure 2D–F). In this way, by controlling the driving current's magnitude and frequency, along with the electric rotating platform's orientation, operators can correspondingly manipulate the magnetic flux density, oscillation frequency, and orientation of the desired magnetic field for precise control over the MBS^2^M's movement.

The MEHA system uses an electric rotating platform to make the steering control of MBS^2^M simpler and more flexible. In addition, this novel system only uses a set of Helmholtz coils as the magnetic field generation device, making the equipment simpler and allowing for the formation of a larger magnetic field in limited space. It also avoids complex calculations with multiple magnetic forces.

### 2.2 Driving Principle

**Figure 3:**
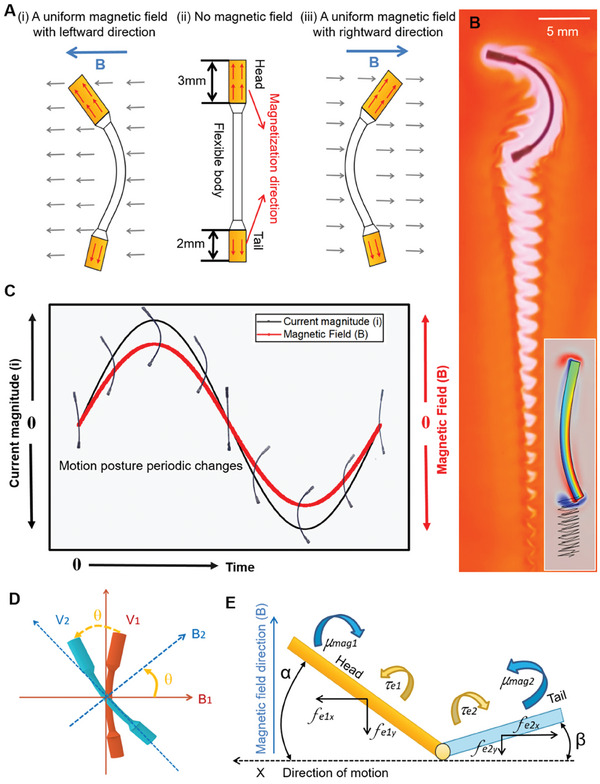
Driving principle of the MBS^2^M. A) Deformation profile of the MBS^2^M under a uniform magnetic field. B) Swimming experiments in an oil film environment and motion simulation (inset). C) Deformation sequence of the MBS^2^M under an oscillating magnetic field. D) Orientation of the MBS^2^M is controlled by modulating the magnetic field direction. E) Analytical model.

The MBS^2^M exhibits swing behavior similar to swimming of crucian carp under the driving of a MEHA system. This swing behavior results from the deformation of the MBS^2^M. The overall appearance of the MBS^2^M is barbell‐shaped and has a special head‐to‐tail proportion structure (with head, tail, and rod lengths of 3, 2, and 10 mm, respectively). The MBS^2^M was fabricated via three cycles of layer deposition using the template‐assisted deposition method.^[^ ^61^, ^62^ ^]^ A detailed structure and more manufacturing details is shown in Figure S2 (Supporting Information). The components at the two ends of the “barbell” had different sizes. We denoted the large end as the head and the small end as the tail. The magnetization orientations of the head and tail were designed opposite along the axial direction. This design facilitates the MBS^2^M to undergo C‐shaped deformation when exposed to a uniform magnetic field. In general, the microrobot maintains a “free” posture without deformation (**Figure** **3**Aii). When a static uniform magnetic field is applied, the head and tail of the MBS^2^M undergo synchronous swing induced by magnetic torque, causing the flexible body to bend into a C‐shape (Figure 3Ai,Aiii). Furthermore, the disparity in magnetic strength between the head and tail causes varying amplitudes of swing. The head, with stronger magnetism, has a larger amplitude of swing, while the tail, with weaker magnetism, has a smaller amplitude of swing. The difference in swing amplitude causes asymmetric flow of the surrounding fluid.

When an oscillating magnetic field is applied, the body of MBS^2^M undergoes periodic deformations in response to the magnetic field, exhibits regular swinging motion similar to the movement of fish. The interaction between the morphological changes of MBS^2^M and the surrounding fluid generates “anti‐Karman vortex” at its tail,^[^ ^66^ ^]^ thereby producing propulsive force to achieve magnetically controlled C‐shaped propulsion movements in a time series. The oil film experiment (Figure 3B and Movie S2, Supporting Information) clearly demonstrates this phenomenon. We also simulated the motion of the microrobot (Figure S3 and Movie S3, Supporting Information) and analyzed the forces on driving force and resistance of robots, for details see the “Motion Simulation” section in Support Information. By modulating the strength and frequency of the external magnetic field through changing excited currents, the motion posture (swing frequency and swing amplitude) of the MBS^2^M exhibited corresponding responsive adjustment (Figure 3C). Additionally, the MBS^2^M's forward direction is controlled by an external magnetic field generated by the MEHA system. The initial motion direction of the MBS^2^M, initial magnetic field direction, adjusted motion direction of the MBS^2^M, and adjusted magnetic field direction are denoted by *V*1,  *B*1,  *V*2,  and *B*2 respectively. As shown in the Figure 3D, the motion direction of the MBS^2^M is consistently perpendicular to the direction of the external driving magnetic field. When the external magnetic field rotates counterclockwise by an angle 𝜃, the MBS^2^M's motion direction will also rotate counterclockwise by the same angle.

To clarify the fundamental movement mechanism of the MBS^2^M, we established an analytical model. Given that the central rod was the longest component and was made of a soft material, the head and tail of the MBS^2^M can be simplified into a rigid slender cylinder and a rigid hollow cylinder, respectively, which were connected by a point joint, as illustrated in Figure 3E. The angles α and β represent the angles between the head/tail and their movement orientation. The joint can be considered as a point hinge. The interaction between the robot and the surrounding fluid results in alterations in body shape, leading to swing swimming motion. The related dynamics can be modeled^[^ ^57^, ^67^, ^68^, ^69^, ^70^ ^]^ as follows:

(1) $$J\left(\theta \right)\ddot{\theta }+C\left(\theta \right){\dot{\theta }}^{2}=D^{\prime }\;\mu_{\mathrm{ela}}+\mu_{\mathrm{mag}}+\tau_{e}+\varphi^{\prime }f_{e},\theta \varepsilon \left(\alpha ,\beta \right)$$

(2) $$m\;\ddot{w}=E^{\prime }f_{e}$$

where J(θ),  and C(θ)εR^2 × 2^ are the matrices of the moment of inertia and centrifugal term, respectively. θεR^2 × 1^is the vector that contains the angular displacement of the two links away from the X‐axis (direction of motion). µ_ela_εR^1 × 1^ is the vector containing recovery torque on the joint generated from the joint elasticity. µ_mag_εR^2 × 1^ is the vector of the magnetic torque at the head and tail of the robot. *f_e_* ∈ *R* ^4 × 1^and τ_e_εR^2 × 1^ are the fluidic forces and torques acting on links, respectively. w ∈ R^2 × 1^is the displacement of the robot on the plane of undulation. φ is the projection matrix, and D and E are two constant matrices. The drag coefficients of fluidic forces and torques can be estimated from the above dynamic equations and experimental data. Thus, the swimming performances of the MBS^2^M can also be predicted. Refer to the supplementary materials for additional details and a discussion of model validation.

### 2.3 Locomotion Performance

**Figure 4:**
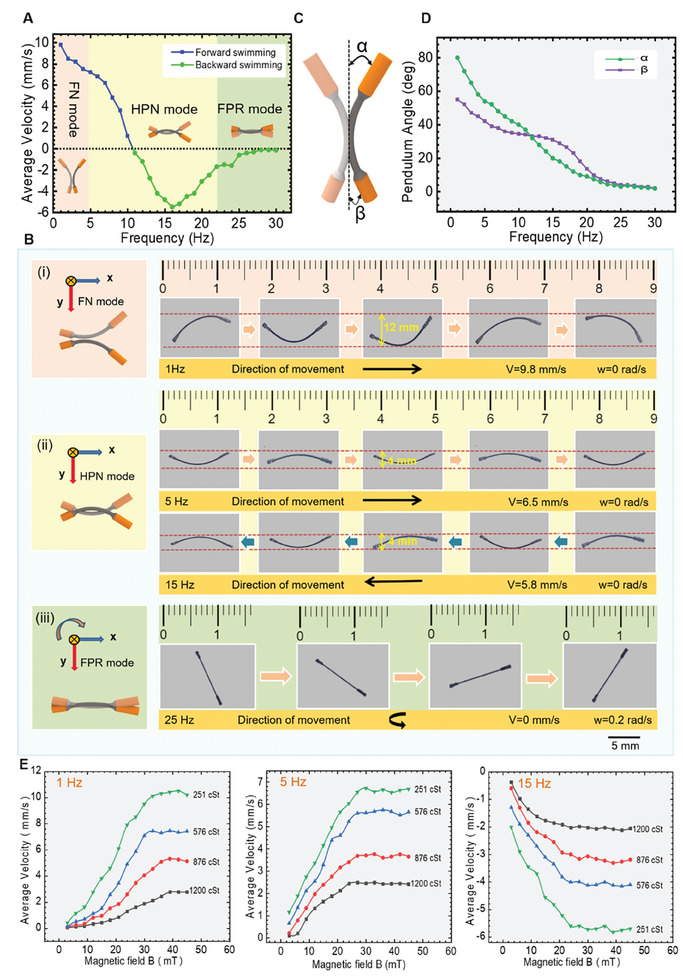
Locomotion performance of the MBS^2^M. A) Experimental data showing the average speed of a MBS^2^M as a function of oscillation magnetic frequency at a field strength of 38 mT. B) Dynamic motion sequences of the MBS^2^M at different frequencies (1, 5, 15, and 25 Hz) under a constant magnetic field of 38 mT. (i) The FN mode. (ii) The HPN mode. (iii) The FPR mode. C) Schematic of the angles between the head and tail of the microrobot and the central axis (α and β). D) The angles (α and β) as a function of the oscillation magnetic field frequency. E) At magnetic field frequencies of 1, 5, and 15 Hz, experimental values of the average speed of the MBS^2^M as a function of magnetic flux density in four different environments. The speed of the MBS^2^M moving to the right is defined as positive velocity.

According to the previous statement, the movement of MBS^2^M can be controlled by varying the magnetic frequency and magnetic flux density. For the analysis of the locomotion performance of the MBS^2^M, we first studied the average velocity of MBS^2^M as a function of different oscillating magnetic frequencies. Here, under the driving of the MEHA system, the motion of MBS^2^M was uniformly characterized in a 90% glycerol solution with a fixed magnetic flux density of 38 mT to exclude the influence of other factors. For simplifying the experimental analysis, the velocity *v* of the microrobot was considered to be relative to the center of the MBS^2^M, and the rotational speed *w* was considered to refer to the motion that is perpendicular to the x‐y plane in which the microrobot body is located. Referring to **Figure** **4**A, we classify the motion patterns into three different mode phases: FN mode, HPN mode, and FPR mode, based on the speed of the MBS^2^M. At low frequencies below 5 Hz (FN mode), the microrobot demonstrated rapid forward motion on the x‐y plane, achieving speeds of up to 9.8 mm s^−1^. As the frequency range shifted to 5 to 25 Hz (HPN mode), the microrobot's speed gradually decreased linearly from 6.5 mm s^−1^. Around 10 Hz, its speed reached zero. With a further increase in frequency, the microrobot moved in the reverse direction without turning and the speed increased linearly. Approximately at 15 Hz, the reverse speed reached 5.8 mm s^−1^. With a further increase in frequency, the reverse movement speed of the microrobot gradually decreased. When the frequency beyond 25 Hz (FPR mode), a further increase in the frequency resulted in a decrease of the speed toward zero. The MBS^2^M cannot produce effective directional movement but only rotate in place following the external magnetic field.

In Figure 4B, representative movement sequences were captured for the three motion phases at distinct oscillation frequencies of 1, 5, 15, and 25 Hz. In the FN mode, the MBS^2^M exhibited significant bending (swing amplitude is about 12 mm) and moved forward rapidly on the x‐y plane (Figure 4Bi and Movie S4, Supporting Information). In the HPN mode, the MBS^2^M exhibited less bending (swing amplitude is about 4 mm) and can move forward or reverse on the x‐y plane (Figure 4Bii and Movie S4, Supporting Information). In the FPR mode, the high‐frequency driving magnetic field generated a small swing amplitude, which prevent effective propulsion and only rotated in a fixed position (Figure 4Biii and Movie S4, Supporting Information). Each of the three motion modes endows the MBS^2^M with unique advantages. In the FN mode, the microrobot rapidly advanced, which can effectively improve its targeted mobility. The HPN mode offers stable movement with high location precision, catering to applications like targeted drug delivery or movement in narrow pipelines. Additionally, forward and backward movement without turning were demonstrated in the HPN mode, which is ideal for navigating narrow channels that can return to the foregone right position when goes the wrong way. The FPR mode enables in‐place rotation, facilitating the microrobot's orientation adjustment to choose the right path to reach the target. By combining the above three basic motion modes, the MBS^2^M can realize excellent motility and adaptability within diverse environments to handle tasks.

The movement of MBS^2^M is a result of the asymmetrical swinging of head and tail. To further understand the locomotion of MBS^2^M, we introduce the parameters α and β, which denote the maximum swing angle between the head and tail of the microrobot and the central axis (Figure 4C). The measured angles α and β as a function of the external oscillation magnetic field frequency were plotted in Figure 4D. When the frequency was below about 10 Hz, α exceeded β, propelling the MBS^2^M forward; In the frequency field range of 10–25 Hz, β surpasses α, resulting in the MBS^2^M's reverse motion; With further frequency increment, α and β converge, causing the MBS^2^M to rotate in a fixed position. Throughout the process, as the frequency of the magnetic field increases, the swing angles (α and β) gradually decreases, which is caused by frequency distortion of MBS^2^M^[^ ^17^ ^]^ in high‐frequency magnetic fields. “Frequency distortion” can be understood as: With the frequency increases, the microrobot's response gradually lags behind the oscillation frequency of the magnetic field because the motion of robots is the actual movement, they require an appropriate response time. As the frequency increases, its swing frequency significantly lags behind the magnetic field's oscillation frequency. Therefore, the microrobot does not have enough time to swing to the corresponding position, leading to a reduced swing amplitude. The reverse motion is essentially due to the different magnetization strengths of the microrobot's head and tail. In a high‐frequency oscillating magnetic field, the difference in magnetization strengths between the head and tail leads to inconsistent levels of “frequency distortion”. This manifests visually as an inversion in the amplitude difference of the head and tail swing. Moreover, we observed that the speed and direction of MBS^2^M is closely related to the difference in swing angles between its head and tail. On the one hand, the greater the angle difference, the faster the movement speed of the microrobot. On the other hand, when the angle difference was positive, the MBS^2^M moved forward; when the angle difference was negative, the MBS^2^M moved reverse.

The magnetic flux density is another crucial parameter that affects the movement of the MBS^2^M. Experimental data illustrating the average speed of the MBS^2^M as a function of magnetic flux density are given in Figure 4E, at magnetic field frequencies of 1, 5, and 15 Hz. Given that MBS^2^M only rotate in place in high frequency magnetic fields (≈25 Hz and above), thereby impeding effective forward motion, we abstain from elaborating on this scenario here. At each magnetic field frequency, the magnetic flux density was modulated from 0 to 45 mT in four liquid environments with different viscosity coefficients (251, 576, 876, and 1200 cSt). The motion posture (swing frequency and swing amplitude) of the MBS^2^M correspondingly adjusted when the liquid viscosity coefficient was the same but the flux density and frequency of the external magnetic field were varied (Figure 3C). The speed of the MBS^2^M gradually increased with the increase in magnetic flux density then subsequently stabilized.

Overall, the above analysis showed that the frequency and flux density of the actuating magnetic field is crucial for regulating the locomotion performance of the MBS^2^M. Capitalizing on these findings and in combination with the advantages of its three basic motion modes, the MBS^2^M can realize effective movement and adaptability across diverse environments.

### 2.4 Navigation Ability

**Figure 5:**
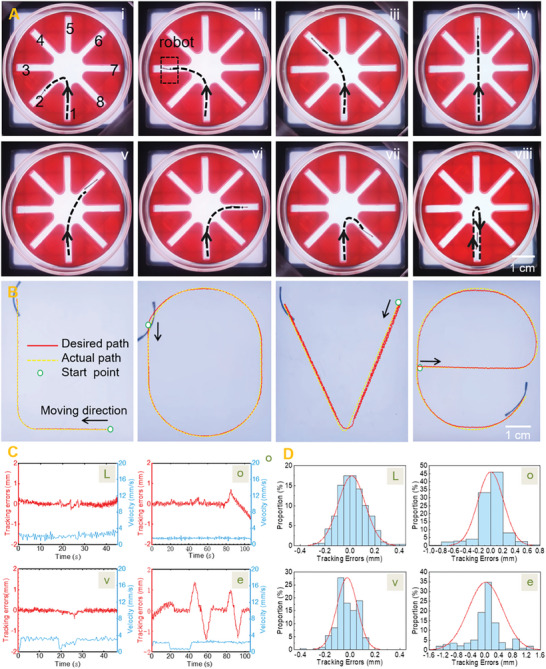
Navigation ability of the MBS^2^M. A) Steering ability experiments. (i) A 135° left turn. (ii) A 90° left turn. (iii) A 45° left turn. (iv) Go straight. (v) A 45° right turn. (vi) A 90° right turn. (vii) A 135° right turn. (viii) A 180° return. The dotted line with arrow represents the MBS^2^M's trajectories. B) A MBS^2^M was actuated to track prescribed trajectories utilizing closed‐loop control. C) Tracking performance results, including tracking errors and real‐time velocity. D) Distribution histogram of the tracking errors. The above experiments were carried out in glycerol with a viscosity of 1200 cSt.

Employing the HPN mode in tandem with the electric rotating platform facilitated the MBS^2^M to not only move accurately but also turn smoothly and flexibly. To substantiate the exceptional navigation performance of the MBS^2^M, an experimental setting containing eight channels was devised (each channel was 30 mm in length, 5 mm in width, and 5 mm in depth), as shown in **Figure** **5**Ai. Adjacent channels were 45° apart. The channels were numbered 1–8 to simplify description. The experiments were divided into three groups and performed eight times (Movie S5, Supporting Information). The MBS^2^M was driven from channel 1 to channels 2–8 in a series of navigation assessments. In the first group, the MBS^2^M was driven to channels 3–7 at a magnetic field frequency of 5 Hz. It performed a 90°left turn (Figure 5Aii), a 45° left turn (Figure 5Aiii), a straight linear motion (Figure 5Aiv), a 45° right turn (Figure 5Av), and a 90° right turn (Figure 5Avi). In the second group, the MBS^2^M was driven to channels 2 and 8 at a magnetic field frequency of 1 Hz. It performed a 145° left turn (Figure 5Ai) and a 145° right turn (Figure 5Avii). Here, the use of a low‐frequency oscillating magnetic field was demonstrated beneficial for the large‐angle steering of the microrobot. In the third group, the MBS^2^M was driven to the center of a channel at a magnetic field frequency of 5 Hz, and the magnetic field frequency was adjusted to 25 Hz, prompting the microrobot to execute a 180° clockwise rotation. The microrobot swung at high frequency and slowly rotated with the magnetic field. After completing a 180° rotation, the magnetic field frequency was adjusted to 5 Hz to drive the microrobot back to channel 1 (Figure 5I). Movie S5 (Supporting Information) shows the comprehensive experimental records. These experiments validated that the proposed MBS^2^M has remarkable localization and steering dexterity within intricate confined environments, including confined passages analogous to blood vessels.

As discussed earlier, the MBS^2^M has a stable motion form and high control precision in the HPN mode. Utilizing a vision‐based closed‐loop controller, the MBS^2^M was actuated to tack “L”, “o”, “v”, and “e” trajectory shapes respectively (Figure 5B and Movie S6, Supporting Information). Please refer to the Figure S4 (Supporting Information) for detailed closed‐loop control principles. Figure 5C,D show real‐time analysis of the MBS^2^M's motion errors and velocities. Although these paths are of complex shapes, the MBS^2^M could still stably follow them with a path deviation error within one tenth of the body length, which has potential significance for targeted delivery.

### 2.5 Multi‐Environment Adaptability

The preceding description demonstrates the proposed MBS^2^M exhibits versatility afforded by its multiple locomotion modes and capabilities, enabling adaptability to multifarious environments.

**Figure 6:**
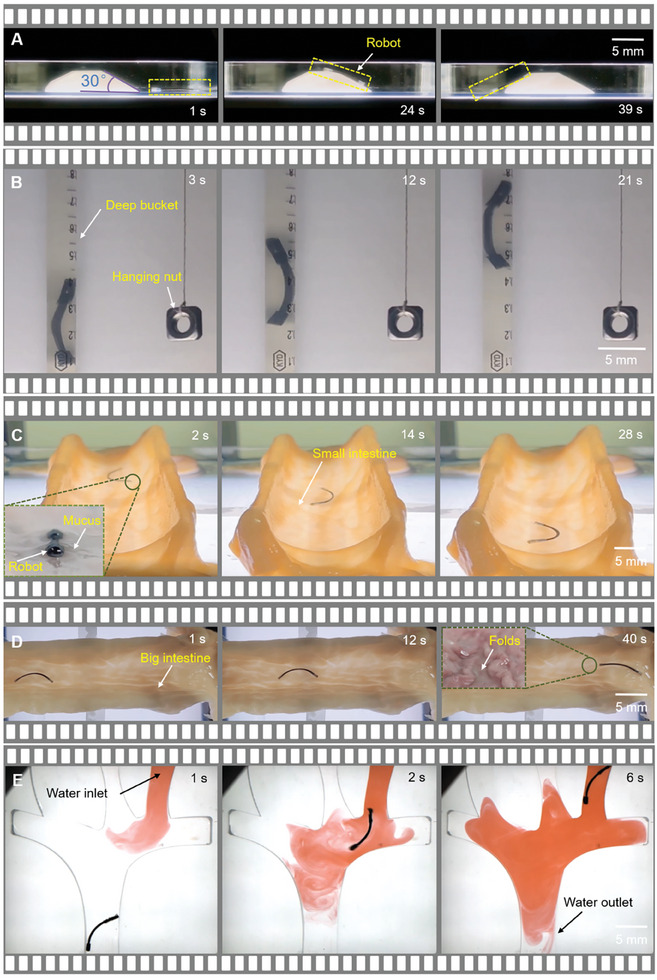
Multi‐environment adaptability. A) Climbing over obstacles with a slope of 30°. B) Overcoming gravity for vertical motion. C) Navigating small intestine mucosal lining. D) Traversing large intestine mucosal folds. E) Traversing in low‐viscosity and low‐flow fluid.

Here, we initiated our investigation by assessing its aptitude for surmounting obstacles. A 3D printed ramp was immersed in 95% glycerol solution to simulate an obstacle in a liquid environment. Under a magnetic field of 1 Hz, the microrobot climbed over the 30° slope successfully (**Figure** **6**A and Movie S7, Supporting Information). The experiment demonstrates the powerful propulsion ability of MBS^2^M. With this ability, under the same experimental conditions, MBS^2^M also can overcome its own gravity to achieve vertical movement in narrow “oceanic trench” analog. This was simulated by suspending a nut in a 4mm‐diameter cylindrical vessel (Figure 6B and Movie S7, Supporting Information). Subsequently, the MBS^2^M was navigated within the mucosal environment of porcine small and large intestines. The small intestine, characterized by a smooth lining (inset in Figure 6C), whereas the large intestine, characterized by a rough lining featuring folds (inset in Figure 6D), both featuring a mucus layer approximately 0.5 mm thick. Employing oscillating magnetic fields with frequencies of 1 and 5 Hz, the MBS^2^M was driven to move within the small intestine (Figure 6C and Movie S7, Supporting Information) and large intestine (Figure 6D and Movie S7, Supporting Information), respectively.

Here, the use of different‐frequency magnetic fields aimed to maximize the microrobots' navigating efficiency in the mucosal milieu. The rough surface of the large intestine posed a challenge to the microrobot's movement, but the MBS^2^M was able to overcome this adversity by utilizing lower‐frequency drives. This adaptive approach resulted in augmented swinging amplitudes of the MBS^2^M's head and tail, effectively enabling it to cover a wider area and navigate terrain limitations. The experiment demonstrated that the MBS^2^M has good movement ability in mucus and can adapt to uneven terrain. Additionally, we conducted fluid experiments in pure water environments. The MBS^2^M demonstrated its ability to overcome resistance in low flow rate conditions (23 mm s^−1^), providing a visual illustration of its performance in low viscosity and low flow fluid environments (Figure 6E and Movie S7, Supporting Information). These abilities further enrich and advance the practical application of MBS^2^M.

### 2.6 Adaptive Locomotion Applied to Targeted Drug Delivery

**Figure 7:**
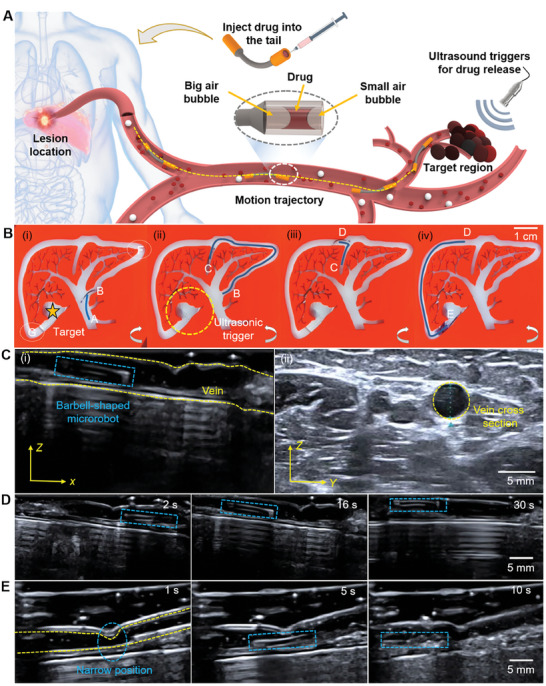
Adaptive locomotion applied to targeted drug delivery. A) Schematic of targeted drug delivery concept. B) Multi‐mode navigation in a liver model containing complex blood vessels. The direction of rotation of the electric rotating platform is represented by a white arrow. C) The vein is marked by the dashed yellow curves in (i) and (ii). Blue box refers to the MBS^2^M. D) Real‐time navigation in ex vivo porcine liver vein. E) Navigating restricted narrow venous regions.

Benefiting from motion stability and extensive adaptability, the MBS^2^M has the potential for performing targeted drug delivery tasks in vivo. As illustrated in **Figure** **7**A and Movie S8 (Supporting Information), a liver model environment containing complex vessels was developed for the simulation of targeted drug delivery. The drug was filled into the inner hollow space of the MBS^2^M's tail. Refer to the “Experimental Section” section for details of the operation. In accordance with previous description, a strategy of multi‐mode control was adopted to drive the MBS^2^M (Figure 7B). When the microrobot was at the starting point A, the space was wide, and the magnetic field frequency was adjusted to 1 Hz to drive the microrobot into the narrow channel quickly in the FN mode (Figure 7Bi). Then, the magnetic field frequency was adjusted to 5 Hz, and the electric rotating platform was rotated anticlockwise to forward drive the microrobot to swing steadily along the channel to the vertical branch channel (Figure 7Bii). The MBS^2^M entered the wrong channel and arrived at point C, while the correct path is through point D. To make the microrobot exit the narrow channel from point C and move back to the desired channel by using the HPN mode, the magnetic field frequency was adjusted to 15 Hz and the electric rotating platform was rotated clockwise (Figure 7Biii). When the microrobot had returned to the desired channel, the frequency of the magnetic field was adjusted to 5 Hz and the electric rotating platform was rotated anticlockwise to forward drive the microrobot to the target point in the HPN mode (Figure 7Biv). Meanwhile, use the FPR mode to help MBS^2^M pass sharp turns at positions F and G. Finally, an ultrasonic instrument was used to trigger drug release. The yellow dashed circle in Figure 7Bii indicates the region triggered by ultrasound. Ultrasonic‐triggered bubble oscillation technology is a novel and feasible method for the remote control of drug release.^[^ ^71^, ^72^, ^73^ ^]^ Please refer to Figure S5 (Supporting Information) for the principles of drug encapsulation and release. The whole process took 182 s, and the average speed reached 2.4 mm s^−1^.

The efficacy of the proposed targeted delivery methodology within blood vessels was subsequently substantiated through an ex vivo navigation study performed in the portal vein of porcine hepatic tissue (Figure 7C). Initially, the MBS^2^M was initially introduced into the vein via a catheter. With the guidance of ultrasound imaging, the navigation of the MBS^2^M in the cross‐sectional (YZ) plane of the portal vein was monitored. By tuning the external driving magnetic field frequency to 5 Hz and intensity to 38 mT, the MBS^2^M was propelled to traverse within the vein. The ultrasound probe was aligned with and trailed the real‐time navigation of the microrobot (Figure 7D and Movie S9, Supporting Information). Subsequently, the capacity of the MBS^2^M to pass through restricted narrow regions of the vein was demonstrated (Figure 7E and Movie S10, Supporting Information). The aforementioned experimentation validated the ability of the proposed MBS^2^M for targeted drug delivery within a complex physiological environment.

## 3 Discussion

**Figure 8:**
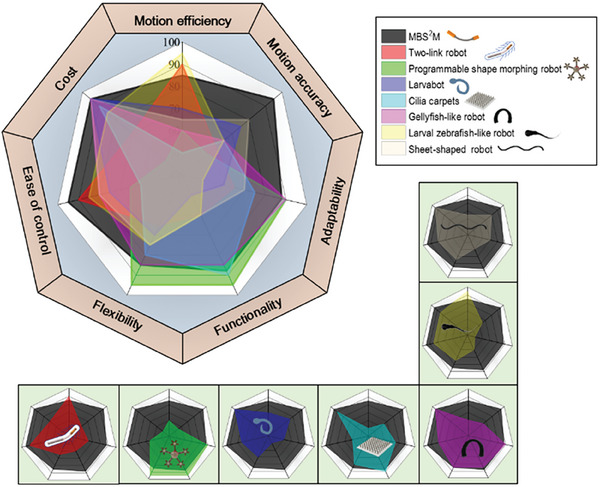
Comparison between MBS^2^M and other f‐MAMRs. Comparison from the aspects of motion efficiency, motion accuracy, adaptability, functionality, flexibility, ease of control, and cost between MBS^2^M and two‐link robot,^[^ ^59^ ^]^ programmable shape morphing robot,^[^ ^58^ ^]^ larvabot,^[^ ^60^ ^]^ cilia carpets,^[^ ^62^ ^]^ jellyfish‐like robot,^[^ ^23^ ^]^ larval zebrafish–like,^[^ ^57^ ^]^ sheet‐shaped robot.^[^ ^61^ ^]^

The development of MAMR, particularly the flexible ones, is driven by the need for minimally invasive and targeted tasks in vivo. To drive the robots reaching the target through complex or confined tubular fluid environments such as blood vessels, is a cornerstone in the field of MAMR, calling for specific MAMR design and magnetic actuation source to realize the environmental adaptative multimodal locomotion. However, motion control in such robots is complex, and current research focuses more on motion in simple liquid environments and limited functionality. Integrating multiple functions and adapting to complex environments like blood vessels remain a challenge. The motion efficiency^[^ ^58^, ^60^ ^]^ and accuracy^[^ ^57^, ^59^ ^]^ of microrobots require improvement, and their adaptability to the environment remains limited. Some microrobots rely on external environmental constraints^[^ ^61^ ^]^ and friction^[^ ^49^, ^62^, ^63^, ^64^ ^]^ for mobility or cannot traverse within narrow spaces.^[^ ^65^ ^]^ In addition, the release of drugs is an ongoing challenge. Compared to previous research, MBS^2^M possesses more functions and has stronger adaptability by ingeniously integrating biomimetic design and a novel magnetic actuation platform. By adjusting the external magnetic field, MBS^2^M can not only switch between three basic motion modes, but also capable of precise steering, obstacle crossing, diverse surfaces moving, reversing without turning, and gravity overcoming. Furthermore, MBS^2^M also has more flexible mobility and accurate positioning ability, enabling precise directional delivery and navigation in complex environments like blood vessels in liver‐model and ultrasound imaging‐monitored ex‐vivo. At the same time, the new MEHA system composed of Helmholtz coils and an electric rotating platform drives the robot in a low‐cost and more flexible manner, providing useful addition for the control of magnetic microrobots. The MEHA system demonstrates high compatibility, also accommodating various driving forms such as swimming, crawling, and helical motion when employed for other robot designs (Movie S11, Supporting Information). We compared the MBS^2^M with several other excellent f‐MAMRs in terms of motion efficiency, motion accuracy, adaptability, functionality, flexibility, ease of control, and cost. Here, a higher score represents a more pronounced advantage in a certain aspect, as shown in **Figure** **8**. Refer to the supplementary materials for additional details.

The proposed MBS^2^M is capable of navigating through challenging environments such as liquid, mucus, and gas‐liquid interface. This could be applied in various biomedical practices such as drug delivery and gastrointestinal surgery. Additionally, the ex‐vivo experiment using blood vessels further validated the capability to pass through narrow passages effectively. However, the human body has even more complex environments with different natural cavities like the brain ventricles,^[^ ^74^ ^]^ urinary tract,^[^ ^75^ ^]^ bile ducts,^[^ ^76^ ^]^ and Eustachian tubes,^[^ ^77^ ^]^ which require robots to adapt to various sizes and possess different magnetic drive systems. Moreover, to achieve more complex tasks like drug delivery, treatment, and obstacle removal, it is also necessary to design robots with different motion modes such as rotation and creep. In addition, biocompatibility, toxicity, and the exclusion or absorption methods of robots in organisms need to be studied for safety consideration.

In order to realize the micromanipulation with high safety, high reliability, and high precision, real‐time image tracking technology of microrobot navigation in complex environment in vivo is needed to be integrated in the proposed system. Ultrasound imaging has the advantage of non‐radiation, but its resolution is relatively low, and it is difficult to distinguish more subtle structures and environments. Photoacoustic imaging is one of the most promising imaging technologies for in vivo applications.^[^ ^78^ ^]^ The interior milieu environment recognition and detection technology based on AI technology will also bring innovations to the navigation and targeted tasks of magnetic field‐controlled microrobots in vivo.^[^ ^79^, ^80^, ^81^ ^]^

## 4 Experimental Section

### Experimental Liquid Environment

In all the above‐mentioned experiments, glycerin aqueous solutions were used to simulate a viscous liquid environment. Glycerin aqueous solutions with different viscosities for each experiment were prepared in accordance with international standards. Refer to the Supplementary Material for additional information (Table S1, Supporting Information). All experiments were carried out at 23 °C in a laboratory environment.

### Oil Film Experiment

First, pour 50 mL of pure glycerol into a square (100 × 100 × 10 mm) container. Then, drop 0.1 mL of red oil onto the surface of the glycerol and let it sit for 5 min. After the red oil has diffused evenly, the MBS^2^M was placed into the oil film for driving.

### Navigation in the Mucus of Porcine Small and Large Intestines

The intestines used in the experiments were taken from pigs and were not subjected to further special treatment to preserve intestinal mucus.

### Navigation in a Liver Model

The liver model used in the experiment was made from resin through 3D printing. For improved visibility, black dye was used to simulate the drug that was being carried and transported by the microrobot. The drug was manually injected into the hollow tail of the MBS^2^M by using an injector. The drug did not spill into the experimental environment due to capillary forces. A large bubble (1.5 mm in length and 0.6 mm in diameter) and a small bubble (1 mm in length and 0.6 mm in diameter) encapsulated the drug in the tail of the MBS^2^M. The large bubble was encapsulated inside the hollow structure of the tail, while the small bubble was encapsulated outside the hollow structure of the tail. This design was beneficial for the subsequent triggering of drug release. Then, drive the MBS^2^M to move in different motion modes in the model. The microrobot moved to the targeted position, and an ultrasound trigger (XJ‐5, Shenzhen Gekeer Trading Co., LTD, China) was used to enable drug release. The principle of drug release was that when the ultrasonic frequency was close to the natural frequency of the large bubble, the large bubble induces large oscillation to expel the drug for release. The ultrasonic trigger (with a power of 70 W and a frequency of 1.9 kHz) used in the experiment was placed at the corresponding point of the targeted position at the bottom of the experimental platform. The above experiments were carried out in 90% glycerol.

### Navigation in Porcine Liver Vein Ex Vivo

A concentration of 2 mg mL^−1^ phosphate‐buffered saline buffer was injected into the porcine liver vein ex vivo to simulate stagnant blood. Ultrasound imaging was conducted using an ultrasound system (Aplio i800, Canon, Japan), with an imaging depth of 3 cm.

## Conflict of Interest

The authors declare no conflict of interest.

## Author Contributions

Q. L., F. N., and H. Y. contributed equally to this work. L.Z., H.Y., and Q.L. conceptualized the study. H.Y., F.N., L.Z., and Q.L. developed the methodology. Q.L., F.N., H.Y., D.X., and C.C. conducted the investigation. J.D. and J.L. performed the ex vivo experiment. F.N., Q.L., and C.C. created the visualization. H.Y., Q.L., D.X., and J.L. analyzed and interpreted the data. Q.L., H.Y., and F.N. wrote the original draft. H.Y., L.Z., and L.S. reviewed and edited the manuscript. L.Z. and L.S. supervised the study.

## Supporting information



Supplemental Material

Supplemental Movie 1

Supplemental Movie 2

Supplemental Movie 3

Supplemental Movie 4

Supplemental Movie 5

Supplemental Movie 6

Supplemental Movie 7

Supplemental Movie 8

Supplemental Movie 9

Supplemental Movie 10

Supplemental Movie 11

## Data Availability

The data that support the findings of this study are available from the corresponding author upon reasonable request.
