# Supplementary material for: Magnetically Actuated Soft Microrobot with Environmental Adaptative Multimodal Locomotion Towards Targeted Delivery
Source: Adv Sci (Weinh). 2024 Sep 24;11(43):2406600. doi: 10.1002/advs.202406600 (PMC11578324; doi:10.1002/advs.202406600)
Supplement: Supplementary file 1 — Supplemental Material [file ADVS-11-2406600-s012.docx]

Supporting Information

**Magnetically actuated soft microrobot with environmental adaptative multimodal locomotion towards targeted delivery**

*Qingwei Li,^1^† Fuzhou Niu,^2^† Hao Yang, ^1^†* Dongqin Xu,^1^ Jun Dai, ^3^ Jing Li, ^4^ Chenshu Chen,^1^ Lining Sun, ^1^* and Li Zhang, ^5^**

**This PDF file includes:**

Supplementary Text

**Other Supplementary Materials for this manuscript include the following:**

Movies S1 to S11

1. Preparation of glycerin aqueous solution with different viscosity

In the experiment of this paper, we tested the MBS^2^M in a liquid environment with different viscosity. Here, glycerin aqueous solution with different densities was used to simulate a liquid environment. All experiments were conducted in an indoor laboratory at 23 ℃.

Table S1. Preparation of different viscosity glycerin aqueous solution

| Viscosity | Density | Specific production method |
| --- | --- | --- |
| 251 cSt | 90% | 90% glycerin，10% water |
| 576 cSt | 95% | 95% glycerin，5% water |
| 876 cSt | 98% | 98% glycerin，2% water |
| 1200 cSt | 100% | 100% glycerin |

2. Oscillating magnetic field

**
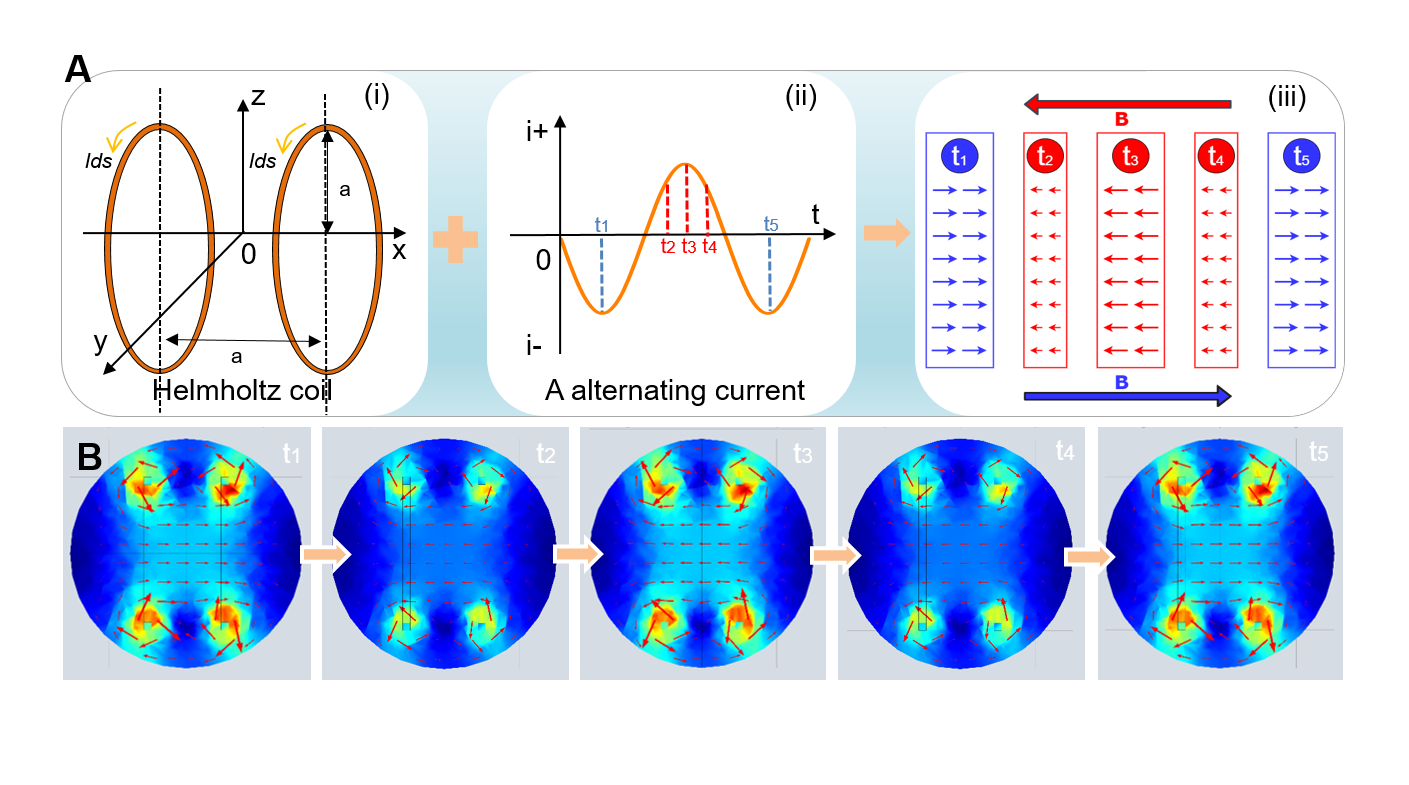
**

**Figure S1. The principle diagram of generating an oscillating magnetic field. (A)** Schematic of the generated oscillating magnetic field. (i) Pair of Helmholtz coils. (ii) Sinusoidal alternating current input. (iii) Schematic of the variation in the generated oscillating magnetic field over time points t_1_–t_5_. **(B)** Simulation of the generated magnetic field over time points t_1_–t_5_.

When a constant current is passed through the Helmholtz coil, a uniform magnetic field can be formed in a small area around the center of the coil. According to the electromagnetic field theory, when a sinusoidal alternating current is applied to the Helmholtz coil and the current changes from t_1_ to t_5_, the induced magnetic field generated is shown in **Figure S1Aiii**. If we define the right direction along the coil axis as the positive direction, the induced magnetic field first decreases in the positive direction, then increases in the negative direction, decreases again, and finally increases in the positive direction. The generated magnetic field is uniform within the workspace at any given moment, whose frequency mirrors that of the excitation current alterations and the magnetic flux density scales proportionally to the current's magnitude. The dynamic simulation of the magnetic field is shown in **Figure S1B** and **Movie S1**.

3. Fabrication and characterization


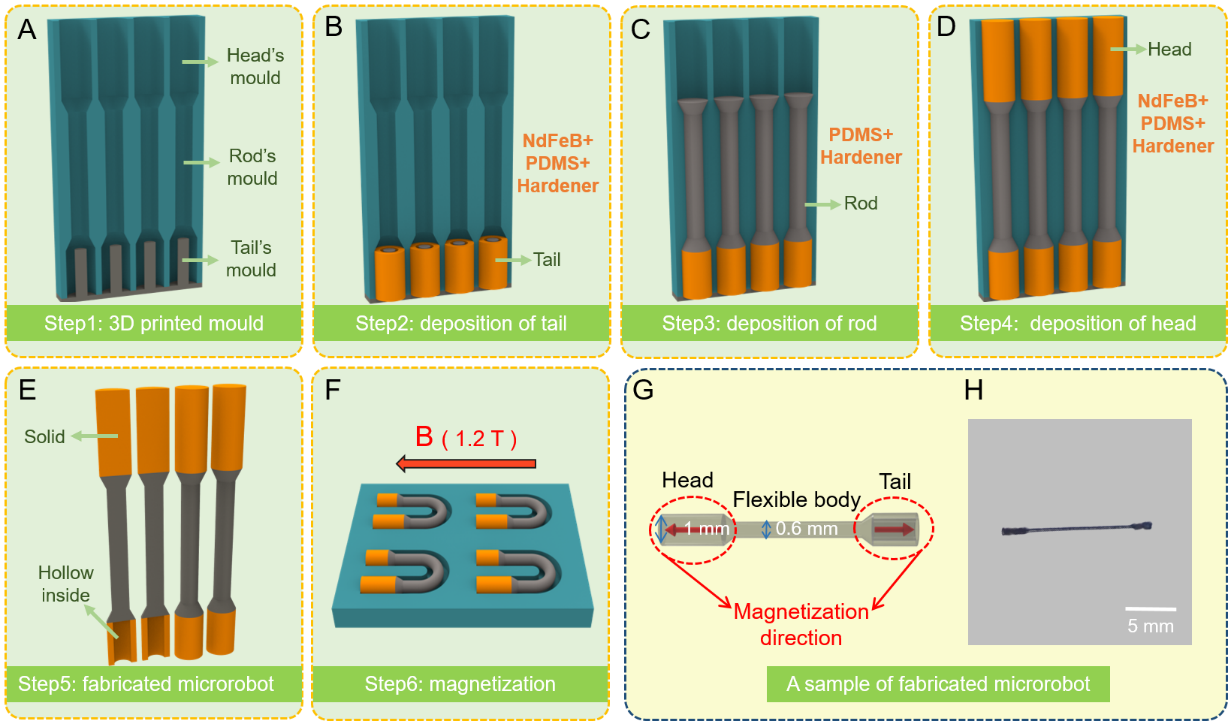


**Figure S2. Manufacture of the magnetic barbell-shaped soft microrobot (MBS^2^M).** (**A**) Preparation of a template for auxiliary deposition. (**B**)-(**D**) The tail, rod and head of the MBS^2^M are obtained by three deposition in sequence. The emulsion (NdFeB+PDMS+Hardener) is filled and remains in the high-aspect-ratio hole structures to make the tail and head of the MBS^2^M. The rod of the MBS^2^M is made of composite materials (PDMS+Hardener). (**E**) Removal of the microrobot from the template. (**F**) The stripped microrobot was placed into a U-shaped template and magnetized in the magnetizer (B = 1.2 T). (**G**) Schematic of the magnetization direction. (**H**) Microscopy image of a fabricated MBS^2^M.

The MBS^2^M was fabricated via three cycles of layer deposition by using the template-assisted deposition method (61, 62). The entire step-by-step manufacturing processes are given and illustrated in **Figure S2**.

Step 1: Preparation of a template for auxiliary deposition. A high-precision 3D printer (UM180, Suzhou Rongzhi 3D Technology Co., LTD, China) was used to print the predesigned mold **(Figure** **S2A)**. The interior of the mold corresponding to the head was empty, whereas the interior of the mold corresponding to the tail contained a cylinder. Thus, the final manufactured tail had a hollow interior, which can be used to load drugs for targeted therapy.

Step 2: First cycle of deposition to fabricate the MBS^2^M tail. NdFeB microparticles (MQP-15-7, Magnequench; average diameter, 5 µm), PDMS (Sylgard 184, Dow Corning), and hardener (Sylgard 184, Dow Corning) were uniformly mixed at a 10:10:1 weight ratio to prepare the tail’s filling emulsion. After the mold was fixed, the emulsion was degassed in a vacuum chamber (BYP-070GX-9ZK, GYPEX, China) and filled into the tail’s mold **(Figure S2B)**. The whole mold was later placed in an oven at 60 °C and baked for 10 min. The emulsion formed a hollow tubular structure due to the presence of the cylinder in the interior of the tail’s mold.

Step 3: Second cycle of deposition to fabricate the MBS^2^M rod. PDMS, hardener, and black dye were uniformly mixed at a 10:1:1 weight ratio to prepare the rod’s filling emulsion. The composite materials was degassed in a vacuum chamber and filled into the rod’s mold **(Figure S2C)**. The whole mold was later placed in an oven at 60 °C and baked for 10 min. Black dye was doped to facilitate the visual detection of the MBS^2^M.

Step 4: Third cycle of deposition to fabricate the MBS^2^M head. The filling emulsion used in this step was the same as that used in Step 2. The emulsion was degassed in a vacuum chamber (BYP-070GX-9ZK, GYPEX, China) and filled into the head’s mold **(Figure S2D)**. Then, the whole mold was placed in an oven at 70 °C for 120 min. During the whole process, the first and second cycles of baking were performed to form the corresponding components’ structures and ensure the strong linkage of each part. The third cycle of baking was performed to solidify the filling material completely to fix the entire structure of the MBS^2^M.

Step 5: After the mold was completely cured and cooled to room temperature, the fixed microrobot was stripped from the mold **(Figure S2E)**.

Step 6: Magnetization of the microrobot. The stripped microrobot was placed into a printed U-shaped groove mold as shown in **Figure S2F**. Then, the U-shaped groove mold was placed into a magnetizer (PFD-1005, Mianyang Litian Magnetoelectric Technology Co., LTD, China) that could generate a magnetic field of ~1.2 T with a direction parallel to the axial line of the head and tail. Given that NdFeB microparticles tend to align their magnetization directions along the external magnetic field, a MBS^2^M whose head and tail were magnetized in opposite directions along the axis was obtained **(Figure S2G)**.

The proposed manufacturing protocol is universal and can be adapted to fabricate MBS^2^M with different configurations and materials. **Figure S2H** presents a microscopy image of a fabricated barbell-shaped microrobot that was ~15 mm in length.

4. Motion simulation

*
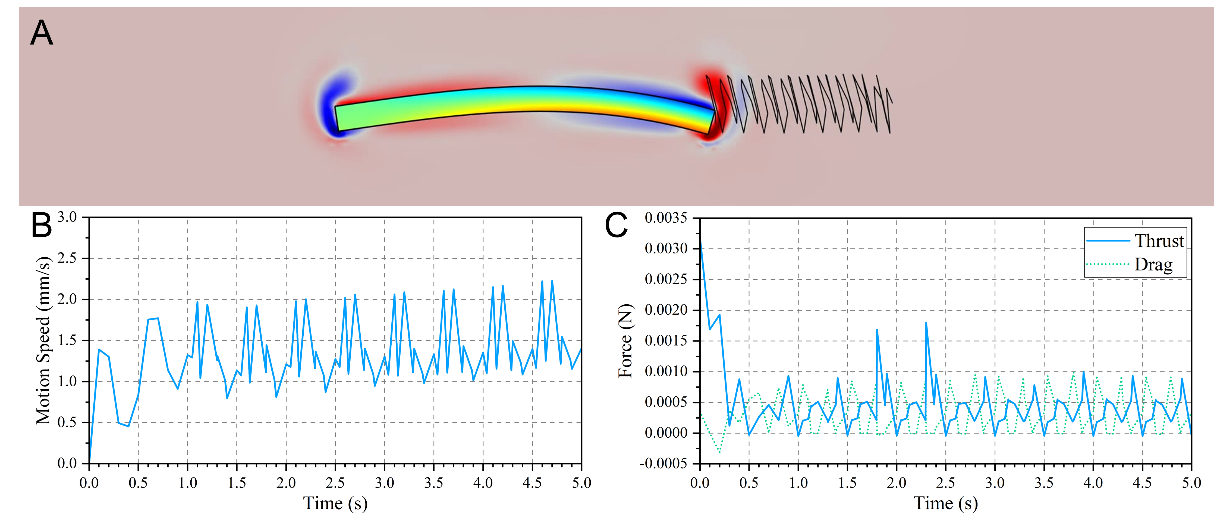
*

**Figure S3. Simulation of robot Movement.** (A) Motion simulation of the MBS^2^M. (B) Motion simulation speed of the MBS^2^M (C) Resistance and driving force value of the MBS^2^M.

To further elucidate the mechanisms of MBS^2^M locomotion, we conducted simulations using COMSOL to model the behavior of MBS^2^M in a static fluid (Figure S3A). The microrobot was simplified to a straight rod shape (length 15 mm, diameter 1 mm). The model parameters were set as follows: magnetic field frequency at 5Hz, magnetic field strength at 5mT, MBS^2^M at 1250 kg/m³, Poisson's ratio of 0.3, fluid environment viscosity at 251cSt, and environment density at 1100 kg/m³. The simulation results, as depicted, include the time-dependent velocity of the MBS^2^M (Figure S3B) and the relationship between the forces of resistance and propulsion of the whole microrobot (Figure S3C).

5. Dynamics modeling

We developed an analytical model to systematically comprehend the theoretical mechanism of the dynamic movement of the MBS^2^M. The magnetic head and tail have been simplified into a rigid slender cylinder link and a rigid hollow cylinder link respectively. These two links are connected by an elastic joint. The clamping angles between the head/tail and their movement orientation respectively are denoted by α and β. The joint has been considered as "point hinges" of zero length to simplify the calculations. Analytical model as shown in **Figure 3E**. Here we frist define several constant matrices used in the dynamics modeling:

$A=\left[ 1 1 \right]$ (S1)

$M=diag\left( m_{1}, m_{2} \right)$ (S2)

$L=diag\left( l_{b1,} l_{b2} \right)$ (S3)

$J=diag\left( J_{1,} J_{2} \right)$ (S4)

$H=LA^{'}\left( AM^{-1}A^{'} \right)\mathrm{AL}$ (S5)

${N=M}^{-1}A^{'}\left( AM^{-1}A^{'} \right)\mathrm{AL}$ (S6)

$E=\left[ \begin{matrix} e & 0 \\ 0 & e \end{matrix} \right]，e={[1, 1]}^{'}\in R^{2\times1}$ (S7)

where $M_{i}$，$l_{\mathrm{bi}}$ and $J_{i}$ are the mass, length, and moment of inertia of each link, respectively. $J_{1}=\frac{md^{2}}{2}$ ，$J_{2}=\frac{m({r_{1}}^{2}+{r_{2}}^{2})}{2}$, where$d$is the radius of the microrobot head,$r_{1}$is the radius of the inner circle of the microrobot tail, $r_{2}$is the radius of the outside circle of the microrobot tail.

The equations of motion governing the forced damped passive swimming of the microrobot can be derived from the first principle of mechanics,^[57, 67-70]^ assuming the microrobot are neutrally buoyant:

$J\left( \theta\right)\ddot{\theta}+C\left( \theta\right)\dot{\theta}^{2}=A^{'}\mu_{\mathrm{ela}}+\mu_{\mathrm{mag}}+\tau_{e}+\varphi^{'}f_{e} , \theta\epsilon\left( \alpha,\beta\right)$ (S8)

$m\ddot{w}=E'f_{e}$ (S9)

Equation S8 describes how the external magnetic torques and fluidic forces lead to the changes in the body shape, and Equation S9 describes how these shape changes further lead to swimming.$J\left( \theta\right),C\left( \theta\right)\in R^{2\times2}$ are the matrices of the moment of inertia, and the centrifugal term, respectively:

$J\left( \theta\right)=J+S_{\theta}HS_{\theta}+C_{\theta}HC_{\theta}$ (S10)

$C\left( \theta\right)=S_{\theta}HC_{\theta}-C_{\theta}HS_{\theta}$ (S11)

$\theta,\dot{\theta},\ddot{\theta}\in R^{2\times1}$ denote the angular displacement, speed, and acceleration of two links, respectively. $\mu_{\mathrm{ela}}\epsilon R^{1\times1}$ is the vector containing elastic torques on joints. According to elementary beam theory, the relationship between the elastic torque and the curvature of a joint can be expressed as:

$\mu_{\mathrm{ela}}=EIK$ (S12)

where$E$ is Young’s modulus of the joint material,$I$ is the second moment of area with$I=\frac{\pi d^{3}}{32}$, and $K$ is the curvature around the joint.$d$is the diameter of the joint, and $K$ can be approximated as:

$K=\frac{2\left| \alpha-\beta\right|}{l_{1}+l_{2}}$ (S13)

where$l_{1},l_{2}$ represents the length of the head and tail of the microrobot.$\mu_{\mathrm{mag}}\in R^{2\times1}$is the vector with the first entry as the magnetic torque applied to the head link and the second entry as the magnetic torque applied to the tail link. The magnetic torque applied on the head and tail link is represented by:

$\mu_{mag1}=T_{m1}=\left| M \right|\left| B \right|\sin({90}^{^{\circ}}-\alpha)$ (S14)

$\mu_{mag2}=T_{m2}=\left| M \right|\left| B \right|\sin({90}^{^{\circ}}-\beta)$ (S15)

$B=\Delta B\sin\left( \omega t \right)$ (S16)

where$M$ is the magnetic moment of the magnetic composite head and tail, $B$ is the external actuation magnetic flux density, $\Delta B$ is the externally driven maximum flux density. $\alpha, \beta$ represents the angle between $M$ and$B$, $f_{e}=\left[ f_{x} ,f_{y} \right]^{'}\in R^{4\times1}$is the vector that contains the decomposed hydrodynamic forces on five links along $x$-axis andy-axis, respectively.$\tau_{e}\in R^{2\times1}$contains the fluidic torques acting on links, respectively. $w\in R^{2\times1}$ is the displacement of the robot on the plane of undulation, $m=\sum_{i=1}^{2} m_{i}$ is the mass of the microrobot. $m_{1}$ is the mass of the head. $m_{2}$is the mass of the tail. $\varphi$ is defined as:

$\varphi=\left[ S_{\theta}N^{'}-C_{\theta}N^{'} \right]^{'}$ (S17)

where

$S_{\theta}=diag\left( \sin\alpha, \sin\beta\right)$ (S18)

$C_{\theta}=diag\left( \cos\alpha, \cos\beta\right)$ (S19)

To compute the hydrodynamic forces, we need one more relation between the individual links’ velocity and the robot’s velocity:

$\dot{Z}=\varphi\dot{\theta}+E\dot{w}$ (S20)

where$Z=\left[ x^{'},y^{'} \right]^{'}\in R^{4\times1}$ is the vector of the displacements of two links stacking together. The velocity of each link can be decomposed to the normal and tangential components $v_{\mathrm{ni}}$ and $v_{\mathrm{ti}}$ respectively. The hydrodynamic force in the normal and tangential directions on the i-th link ($f_{ni}$and$f_{ti}$respectively) can be then modeled based on the resistive force theory.

$f_{ti}=2.7c_{t}l_{bi}\sqrt{p_{w}\mu_{w}w_{b}\left| v_{ni} \right|v_{ti}}$ (S21)

$f_{ni}=sgn\left( v_{ni} \right)c_{p}\left( \frac{p_{w}l_{bi}w_{b}}{2} \right){v_{ni}}^{2}$ (S22)

where$p_{w}$ and $\mu_{w}$are the density and dynamic viscosity of the water at the average water temperature of 23 ℃ in our experiment tank,$w_{b}$is the diameter of the body link,$c_{t}$ and$c_{p}$are drag coefficients in the normal (pressure drag) and tangential directions. The function of $sgn( )$ extracts the sign of a real number. Through coordinate transformation, the relations between${(v}_{xi} ,v_{yi})$and $(v_{ti} ,v_{ni})$can be expressed as:

$v_{ti}=- v_{xi}\cos\theta-v_{ni}\sin\theta, \theta\in\left( \alpha,\beta\right)$ (S23)

$v_{ni}= v_{xi}\sin\theta-v_{yi}\cos\theta,\theta\in\left( \alpha,\beta\right)$ (S24)

Furthermore, the relations between ${(f}_{xi} ,f_{yi})$ and ${(f}_{ti} ,f_{ni})$ can be expressed as:

$f_{xi}= f_{ti}\cos\theta-f_{ni}\sin\theta, \theta\in\left( \alpha,\beta\right)$ (S25)

$f_{yi}= f_{ti}\sin\theta-f_{ni}\cos\theta, \theta\in\left( \alpha,\beta\right)$ (S26)

Finally, the fluidic torque on each link can be modeled as:

$\tau_{i}=-\frac{2}{3}c_{p}p_{w}w_{b}\left( \frac{l_{i}}{2} \right)^{3}\left| v_{ni} \right|\dot{\theta}, \theta\in\left( \alpha,\beta\right)$ (S27)

Estimation of the drag coefficients$\boldsymbol{c}_{\boldsymbol{t}}$ and$\boldsymbol{c}_{\boldsymbol{p}}$

The robotic swimmers operate within a wide range of the intermediate flow regime, which means that the drag coefficients are not constant but vary with the local Reynolds Number (Re). Instead of using two fixed drag coefficients, we assume a linear relationship between the local Re and the drag coefficients. For each link$i$, the relation can be expressed as:

$c_{p}=a_{p}\left| \mathrm{Re}_{ni} \right|+b_{p}$ (S28)

$c_{t}=a_{t}\left| \mathrm{Re}_{ti} \right|+b_{t}$ (S29)

where $\mathrm{Re}_{ni}$ and $\mathrm{Re}_{ti}$ are calculated from the local speeds that are normal and tangential to each link, respectively:

$\mathrm{Re}_{ni}=\frac{p_{w}v_{ni}l_{bi}}{\mu w}$ (S30)

$\mathrm{Re}_{ti}=\frac{p_{w}v_{ti}l_{bi}}{\mu w}$ (S31)

The values of $a_{p},b_{p},a_{t}$ and $b_{t}$ can be obtained through experimental test.

6. Position-based closed-loop feedback control


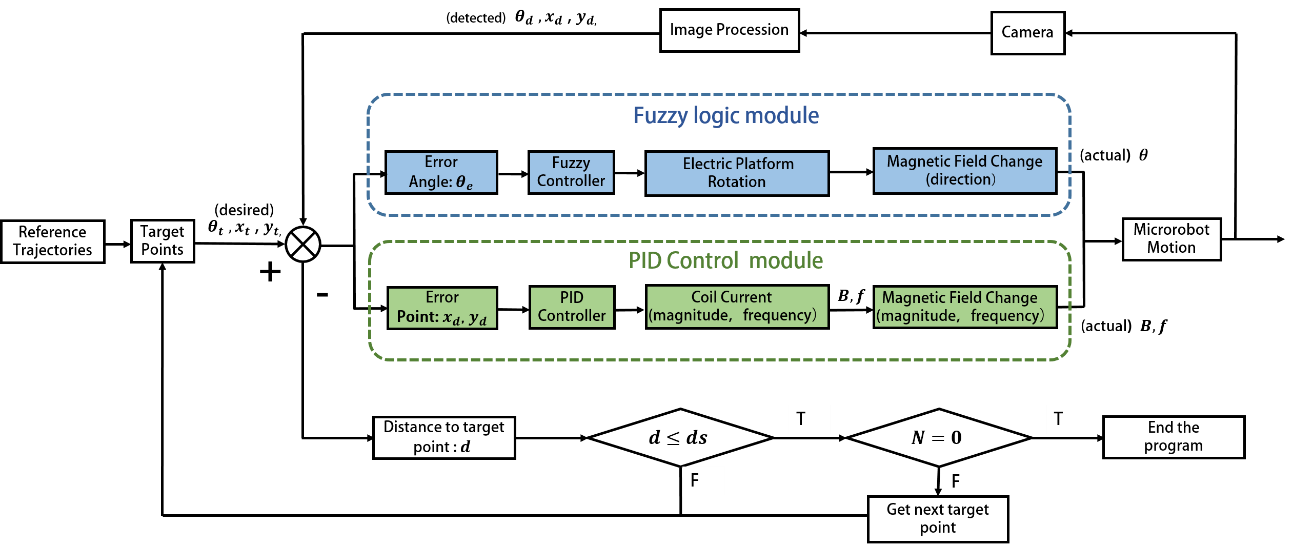


**Figure S4. Position-based closed-loop feedback control**

The MEHA system is based on position feedback and uses a combination of PID and fuzzy algorithms for closed-loop control of the MBS^2^M's motion. In the trajectory tracking experiment mentioned above, we first extract a series of key target points from a complex reference path. Then, the MBS^2^M was driven to reach each target point one by one. In this way, the tracking of the entire complex path is decomposed into simple control of a series of target points, as shown in **Figure S4**. To improve the accuracy of MBS^2^M driving, we use visual detection technology to acquire real-time position information ($\theta_{d},x_{d},y_{d}$) of the robot during the control process. We compare this information with the position information of the target point ($\theta_{t},x_{t},y_{t}$) to obtain position error ($\theta_{e}$) and direction error ($x_{e},y_{e}$). Then, based on the error information, we employ the PID algorithm and fuzzy algorithm for closed-loop control to adjust the motion posture and direction of the MBS^2^M. Specifically, based on the position error information, we use the PID algorithm to adjust the current and frequency in the input coil, thereby controlling the magnetic induction intensity and oscillation frequency of the driving magnetic field and further controlling the motion posture of the MBS^2^M. Secondly, based on the direction position error information, we use the fuzzy algorithm to control the steering of the electric turntable, adjusting the direction of the driving magnetic field and thus controlling the motion direction of the MBS^2^M. Execute the above steps for iteration to achieve autonomous control of robot trajectory tracking. Simultaneously, calculate the distance (*d*) between the robot and the current target point. If this distance ($d$) exceeds the preset threshold (*ds*), then keep the current point as the target point; otherwise, the program will check the remaining number of target points. If the number of remaining target points ($N$) is zero, it indicates that the robot has reached the vicinity of the endpoint, and the program ends immediately; otherwise, select the next target point as the new target, and the program restarts to repeat the entire trajectory tracking until it is automatically completed.

7. Drug encapsulation and release


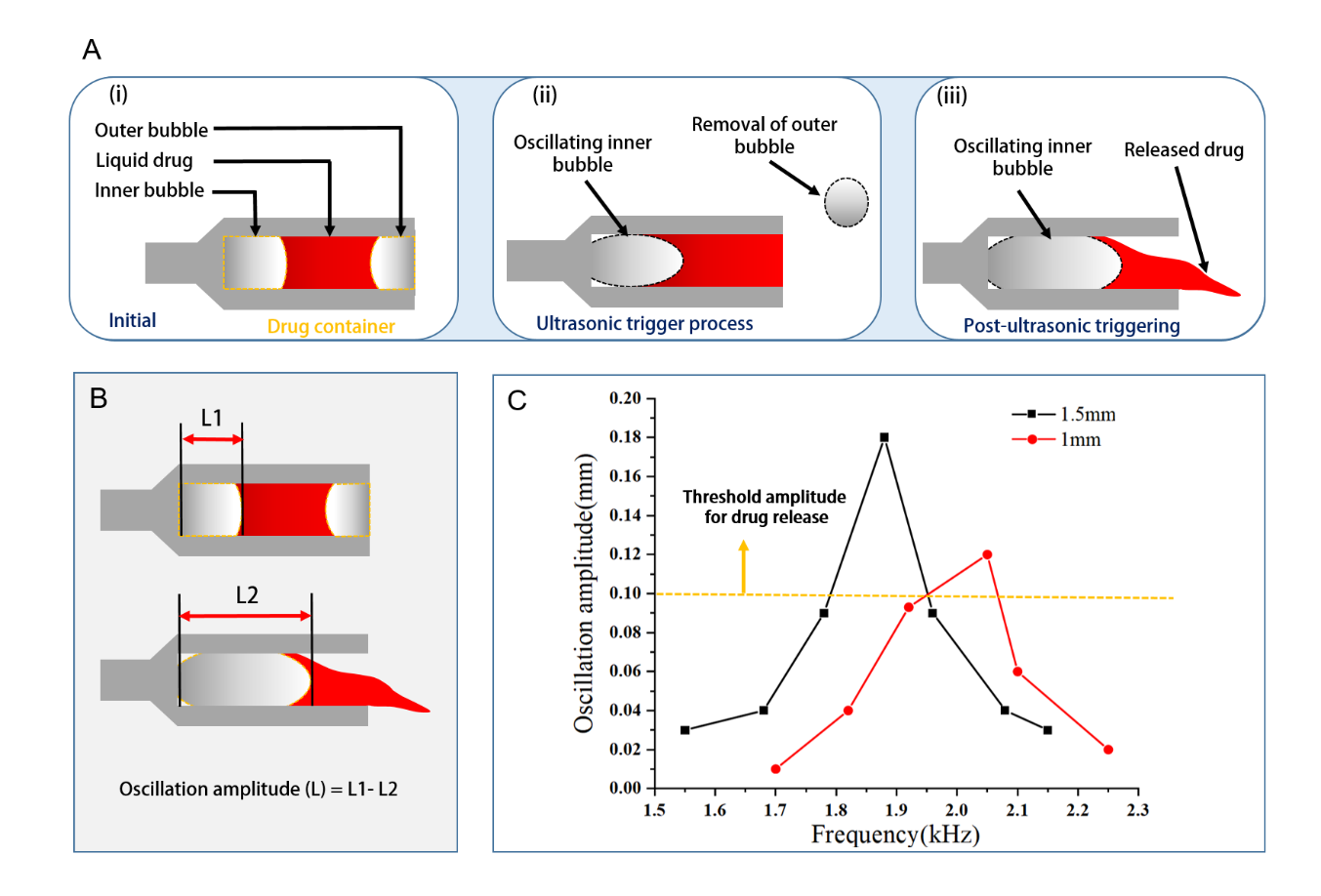


**Figure S5. Drug encapsulation and release. (A)** Schematic diagram of drug encapsulation and release **(B)** Definition of bubble oscillation amplitude. **(C)** Measured oscillation amplitudes of acoustically excited bubbles

(1, and 1.5 mm length) in different frequencies.

The encapsulation and release of drugs are crucial steps in targeted drug delivery of MBS^2^M. In this paper, we utilized differently sized bubbles to encapsulate liquid drugs in the microtubular structures at the tail of MBS^2^M. When manually injecting drugs into the middle of the microtube using a microinjector, a large bubble (1.5 mm in length and 0.6 mm in diameter) automatically becomes trapped between the drug and the closed end of the microtube. When the microtube containing the liquid drug is submerged in an aqueous medium, a small bubble isolating the liquid drug from the aqueous medium is naturally trapped at the opening of the microtube owing to the hydrophobic inner surface of the microtube. Consequently, the drug is encapsulated by the two gaseous bubbles in the microtube. Large bubbles are on the inside, small bubbles are on the outside, as shown in **Figure S5A**. This design facilitates subsequent ultrasound-triggered drug release. When the inner bubble in the drug container is acoustically excited around its natural frequency, the outer bubble is pushed out of the microtube owing to the oscillation of the excited inner bubble, resulting in the opening of the container. Sequentially, the liquid drug is emitted to the aqueous medium to achieve drug release. In addition, based on previous research,^[71]^ we measured the oscillation amplitude of two different lengths of bubbles (1mm and 1.5mm) at different frequencies **(Figure S5C)**. Here, the oscillation amplitude is defined as the difference in length (L) between the maximum expansion of the bubble and its initial state, as shown in **Figure S5B**. The maximum amplitude of bubble oscillation depends on the bubble length and the frequency of the sound wave. The experimental findings indicate that the threshold amplitude for drug release is approximately 0.1mm. Longer bubbles have larger amplitudes compared to shorter bubbles, making it easier to reach the threshold for drug release. Therefore, in the experiment, we chose to use ultrasound with a frequency of 1.9 kHz to trigger drug release.

**8. MBS^2^M and other f-MAMRs Performance index**

We have compared the performance of MBS^2^M with other flexible microrobots. First, we selected representative flexible microrobots from recent years. Subsequently, we compared and scored them based on their performance in motion efficiency, motion accuracy, adaptability, functionality, flexibility, ease of fabrication, and cost. The scoring was primarily based on original data provided in published articles and supplementary materials. The evaluation criteria for each indicator are shown in **Table S2**. We have compiled and summarized the original data for each robot, as shown in **Table S3**. Through comparison, we conducted ranking and scoring, with scores ranging from 60 to 95 on a percentage scale. At the same time, we have improved the supplementary information.

**Table S2. Microrobot evaluation standard index**

| **Indicators** | **Motion efficiency** | **Motion accuracy** | **Adaptability** | **Functionality** |
| --- | --- | --- | --- | --- |
| **Evaluation Criteria** | The motion speed of the microrobot is recorded in the unit of body length. For example, the body length of the robot is 15mm and the movement is 15mm per second, that is, 1 body length/s | Motion accuracy refers to the size of motion error. If there is no relevant test data, the qualitative estimation is based on the motion video | Based on the environments in which robots adapt, the following six scenarios can be identified:  1. Confined liquid environment  2. Unconfined liquid environment  3. Confined solid surface  4. Unconfined solid surface  5. Confined solid-liquid interface  6. Unconfined solid-liquid interface | It is evaluated from two perspectives:  1. The type of motion mode  2. Application of robot |
| **Indicators** | **Flexibility** | **Ease of control** | **Cost** |  |
| **Evaluation Criteria** | It is evaluated from two perspectives:  1. The degree of flexibility of the overall structure of the robot  2. Microrobot deformability | It is evaluated from two perspectives:  1. Number of parameters for robot Motion Control  2. Control Form: Open-loop or Closed-loop Control | It is evaluated from two perspectives:  1. Robot production process  2. Magnetic drive form |  |

Table S3. The original data of microrobot indicators

|  | **Motion efficiency** | **Motion**  **accuracy** | **Adaptability** | **Functionality** | **Flexibility** | **Ease of control** | **Cost** |
| --- | --- | --- | --- | --- | --- | --- | --- |
| **Barbell-shaped soft microrobot** | ≈ 0.65 body length/s | error within one tenth of the body length | 1. Confined liquid environment  2. Unconfined liquid environment  3. Confined solid-liquid interface  4. Unconfined solid-liquid interface | 1. Fast navigation  2. High-precision navigation  3. Fixed-point rotation  4. Reverse swimming  5. Climbing uphill  6. Movement in mucus  7. Targeted drug delivery  8. Movement in low viscosity and low velocity fluids | Flexibility of the Body  1. Overall Flexibility  Produce swing deformation and can be adjusted | Control Parameters  1. Current Frequency  2. Current Intensity  3. Rotating table rotation angle  Control Modes  1. Closed-Loop Control | Fabrication process  1.General template-assisted deposition  Driving magnetic field  1.Homemade mechano-electromagnetic hybrid actuation (MEHA) system |
| **Two-link robot**^[59]^ | ≈ 3.5 body length/s | No tests are done, only the motion trajectories are shown | 1. Unconfined liquid environment | 1. Tumbling  2. Helical swimming  3. Asynchronous swimming  4. Drug load and release | Flexibility of the Body  1. Partial Flexibility（Rigidity of head and tail）  Produce helical deformation and can be adjusted | Control Parameters  1. Current Frequency  2. Current Intensity  3. Current Phase Angle  Control Modes  1. Open-Loop Control | Fabrication process  1.Template-assisted electrodeposition  Driving magnetic field  1.Rotating magnetic field |
| **Programmable shape morphing**  **robot**^[58]^ | No tests have done | No tests have done, only the motion trajectories are shown | 1. Confined liquid environment  2. Unconfined liquid environment  3. Confined solid surface  4. Unconfined solid surface  5. Confined solid-liquid interface  6. Unconfined solid-liquid interface | 1. Temperature sensing  2. PH sensing  3. Multidirectional UV light sensing  4. Circuit repairing  5. Gastric ulcer coating  6. Multimodal motion  7. Oil detection | Flexibility of the Body  1. Overall Flexibility  Perform 2D and 3D programmable deformation | Control Parameters  1. Direction of Magnetic field  2. Magnetic field gradient  Control Modes  It was not explicitly mentioned | Fabrication process  1.Template-assisted method (2D /3D programmable)  2.Multiple module integration  Driving magnetic field  1.Gradient Magnetic Field  2.Permanent Magnet |
| **Larvabot**^[60]^ | ≈ 1.5 body length/s | No tests have done, only the motion trajectories are shown | 1. Unconfined liquid environment  2. Small size tube | 1. Move in 3D Liquid Space | Flexibility of the Body  1. Overall Flexibility  Deform into arc shapes by applying static magnetic fields | Control Parameters  1. Current Frequency  2. Current Intensity  3. Direction of Magnetic Field  Control Modes  It was not explicitly mentioned | Fabrication process  1.General template-assisted deposition  Driving magnetic field  2.Rotating magnetic field |
| **Gilia carpets**^[62]^ | ≈ 0.02 body length/s | No tests have done, only the motion trajectories are shown | 1. Confined solid surface  2. Unconfined solid surface | 1. Rolling  2. Crawling  3. Carry objects | Flexibility of the Body  1. Overall Flexibility  Crawling and Rolling with Programmable Cilia and Soft Body | Control Parameters  1. Direction of Magnetic Field  2. Intensity of Magnetic Field  3. Angular Velocity of Magnetic Field  Control Modes  1. Open-Loop Control | Fabrication process  1.Template-assisted Deposition  Driving magnetic field  2.Rotating Magnetic Field |
| **Jellyfish-like robot**^[23]^ | ≈ 0.15 body length/s | No tests have done, only the motion trajectories are shown | 1. Confined solid surface  2. Unconfined solid surface  3. Unconfined liquid environment  4. Confined solid-liquid interface  5. Unconfined solid-liquid interface | 1. Rolling  2. Crawling  3. Swimming  4. Meniscus climbing  5. Landing  6. Immersion  7. Walking  8. Jumping | Flexibility of the Body  1. Overall Flexibility  Rolling and Diving, Surface Swimming, Meniscus Climbing, Jumping, Walking, Emersion, Immersion | Control Parameters  1. Direction of Magnetic Field  2. Intensity of Magnetic Field  Control Modes  It was not explicitly mentioned | Fabrication process  1.Template-assisted Method  Driving magnetic field  2.Spatial Magnetic Field |
| **Larval zebrafish-like robot**^[57]^ | ≈ 13.2 body length/s | No tests are done, only the motion trajectories are shown | 1. Unconfined liquid environment | 1. Fast swing | Flexibility of the Body  1. Overall Flexibility  Produce swing deformation and can be adjusted in the oscillating magnetic field | Control Parameters  1. Current Frequency  2. Current Intensity  3. Magnetic field direction  Control Modes  1. Closed-Loop Control | Fabrication process  1.Two-step Template-assisted Molding  Driving magnetic field  2.Oscillating Magnetic Field  (Three-axis Helmholtz Electromagnetic Coils) |
| **Sheet-shaped**  **robot**^[61]^ | ≈ 1.08 body length/s | Lack of motion skills display | 1. Restricted liquid environment  2. Restricted fluid environment | 1. Rolling  2. Undulatory crawling  3. Undulatory swimming  4. Helical surface crawling | Flexibility of the Body  1. Overall Flexibility  Produce rolling, crawling and swim, helical crawling deformation | Control Parameters  1. Current Frequency  2. Current Intensity  3. Current Phase Angle  Control Modes  1. Open-Loop Control | Fabrication process  1.laser cutting  Driving magnetic field  Rotating magnetic field |

**References:**

| 23. | W. Hu, G. Z. Lum, M. Mastrangeli, M. Sitti, Small-scale soft-bodied robot with multimodal locomotion. *Nature*. **554**, 81–85 (2018). |
| --- | --- |
| 57. | T. Wang, Z. Ren, W. Hu, M. Li, M. Sitti, Effect of body stiffness distribution on larval fish-like efficient undulatory swimming. Sci. Adv. **7** (2021), doi:10.1126/sciadv.abf7364. |
| 58. | Y. Dong, L. Wang, N. Xia, Z. Yang, C. Zhang, C. Pan, D. Jin, J. Zhang, C. Majidi, L. Zhang, Untethered small-scale magnetic soft robot with programmable magnetization and integrated multifunctional modules. *Sci. Adv.* **8**, eabn8932 (2022). |
| 59. | J. Wu, B. Jang, Y. Harduf, Z. Chapnik, Ö. B. Avci, X. Chen, J. Puigmartí-Luis, O. Ergeneman, B. J. Nelson, Y. Or, S. Pané, Helical klinotactic locomotion of two-link nanoswimmers with dual-function drug-loaded soft polysaccharide hinges. *Adv. Sci. (Weinh.)*. **8**, 2004458 (2021). |
| 60. | N. Xia, B. Jin, D. Jin, Z. Yang, C. Pan, Q. Wang, F. Ji, V. Iacovacci, C. Majidi, Y. Ding, L. Zhang, Decoupling and reprogramming the wiggling motion of midge larvae using a soft robotic platform. Adv. Mater. 34, e2109126 (2022). |
| 61. | Z. Ren, R. Zhang, R. H. Soon, Z. Liu, W. Hu, P. R. Onck, M. Sitti, Soft-bodied adaptive multimodal locomotion strategies in fluid-filled confined spaces. *Sci. Adv.* **7**, eabh2022 (2021). |
| 62. | H. Gu, Q. Boehler, H. Cui, E. Secchi, G. Savorana, C. De Marco, S. Gervasoni, Q. Peyron, T.-Y. Huang, S. Pane, A. M. Hirt, D. Ahmed, B. J. Nelson, Magnetic cilia carpets with programmable metachronal waves. *Nat. Commun.* **11**, 2637 (2020). |

9. Description of video content

Movie S1.

Dynamic simulation of oscillating magnetic field

Movie S2.

Locomotion in an oil film environment

Movie S3.

Motion simulation

Movie S4.

Three basic motion modes of MBS^2^M

Movie S5.

Multi-angle steering test of MBS^2^M

Movie S6.

The MBS^2^M walks according to the predetermined route

Movie S7.

Locomotion in multiple environments

Movie S8.

Targeted drug delivery in a liver model

Movie S9.

Navigation of MBS^2^M in the hepatic vein ex vivo

Movie S10.

Through the narrow position of the portal vein in the porcine liver

Movie S11.

The MEHA system drives microrobots to realize various forms of motion
